# Supplementary material for: Controlled and Sequential Delivery of Stromal Derived Factor-1 α (SDF-1α) and Magnesium Ions from Bifunctional Hydrogel for Bone Regeneration
Source: Polymers (Basel). 2022 Jul 15;14(14):2872. doi: 10.3390/polym14142872 (PMC9315491; doi:10.3390/polym14142872)
Supplement: Supplementary file 1 [file polymers-14-02872-s001.zip › polymers-1782919-supplementary.pdf]

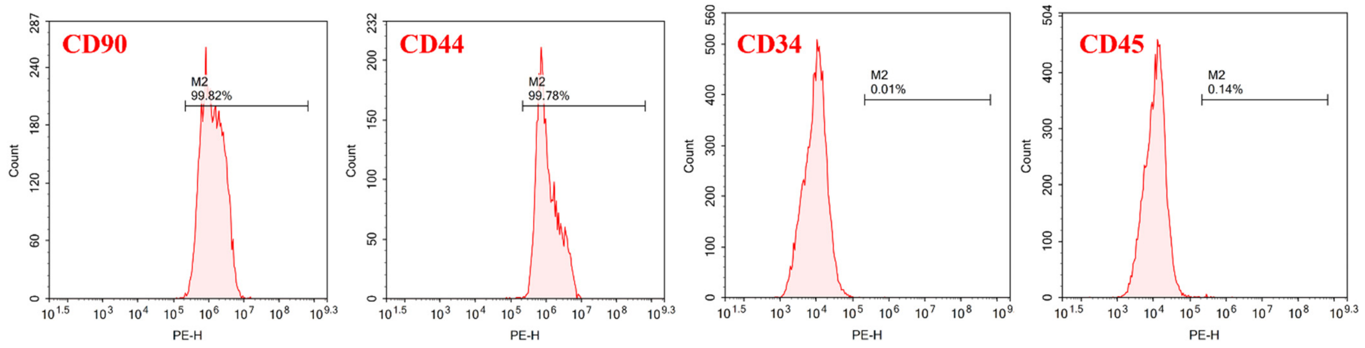

Figure S1. Identification of BMSCs in flow cytometry.

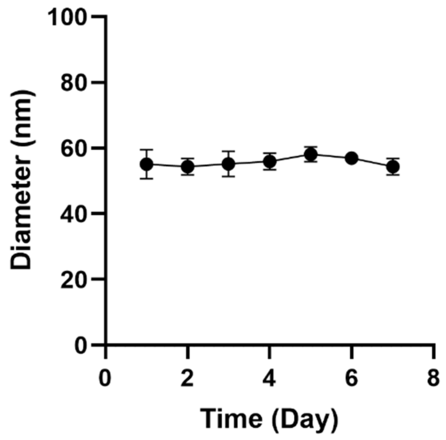

Figure S2. Particle size variation of Mg-NPs for 7 days

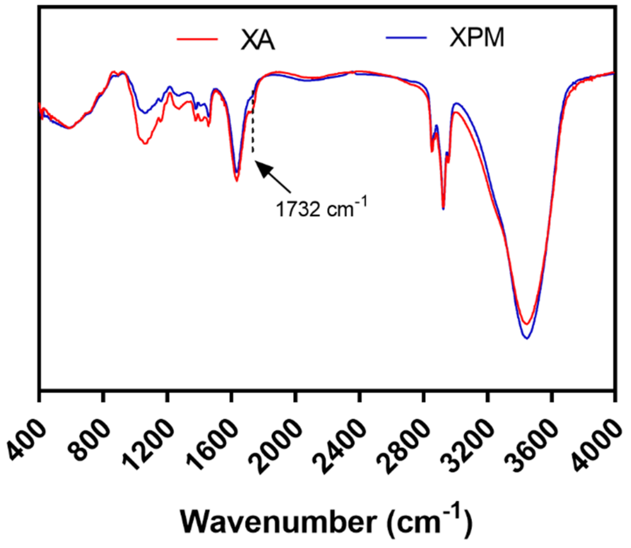

Figure S3. IR spectra of XA and XPM.

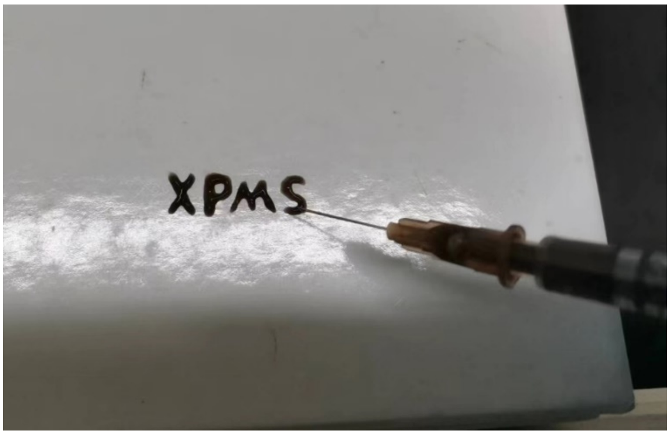

Figure S4. Injectable properties of XPMS hydrogel

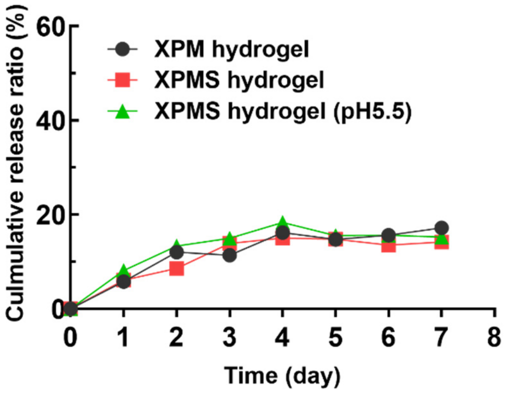

Figure S5. Cumulative release ratio of Mg^{2+} from the XPMS hydrogel under different conditions in vitro.

Abbreviations:

SDF-1α, Stromal derived factor-1α; CXCL12, C-X-C Motif Chemokine Ligand 12; CXCR4, C-X-C motif chemokine receptor 4; Mg^{2+}, Magnesium ions; PDA, polydopamine; Mg-NPs, Mg^{2+} included nanoparticles; XG, xanthan gum; Xan-CHO, aldehyde xanthan gum; SEM, scanning electronic microscope; TEM, Transmission Electron Microscope; EDS, Energy Dispersive Spectroscopy; BMSCs, bone mesenchymal stem cells; XA, pure xanthan gum; XP, polydopamine modified xanthan gum; XS, SDF-1α included pure xanthan gum; XPS, SDF-1α included polydopamine modified xanthan gum; XPM, Mg^{2+} included polydopamine modified xanthan gum; XPMS, SDF-1α and Mg^{2+} included polydopamine modified xanthan gum; ALP, Alkaline phosphatase; ARS, alizarin red staining; BV/TV, bone volume fraction; Tb.N, trabecular number; Tb.Sp, trabecular separation
